# Fabrication of Zr_2_WP_2_O_12_/ZrV_0.6_P_1.4_O_7_ composite with a nearly zero-thermal-expansion property

**DOI:** 10.1016/j.matlet.2017.07.051

**Published:** 2017-11-15

**Authors:** Ikuo Yanase, Hiroshi Sakai, Hidehiko Kobayashi

**Affiliations:** Saitama University, Faculty of Engineering, Department of Applied Chemistry, 255 Shimoohkubo, Sakura, Saitama, Saitama 338-8570, Japan

**Keywords:** Ceramics, Composite materials, Thermal expansion, Sintering

## Abstract

Sintered bodies of Zr_2_WP_2_O_12_ (ZWP) and ZrV_0.6_P_1.4_O_7_ (ZVP) were fabricated, and their linear thermal expansion coefficients (TEC) were found to be −2.92 × 10^−6^ and 3.27 × 10^−6^ °C^−1^, respectively, in the range 25–500 °C. In an attempt to fabricate composites with a zero-thermal-expansion property, sintered ZWP/ZVP composites with ZVP/ZWP volume ratios of 0.5/0.5, 0.53/0.47, 0.55/0.45, and 0.6/0.4 were fabricated. Scanning electron microscopy revealed that sintering of ZVP/ZWP composites progressed well compared with that of ZWP. A porous ZVP/ZWP composite with a relative density of ca. 83% was fabricated at a ZVP/ZWP volume ratio of 0.53/0.47. X-ray diffractometry and energy dispersive X-ray spectrometry clarified that the ZVP/ZWP composite mainly consisted of ZWP and ZVP grains. Thermomechanical analysis confirmed that the ZVP/ZWP composite exhibited very low thermal expansion with a slight hysteresis with a TEC of −0.29 × 10^−7^ °C^−1^ in the range 25–500 °C.

## Highlights

• The sintered ZrV_0.6_P_1.4_O_7_ (ZVP)/Zr_2_WP_2_O_12_ (ZWP) composites were fabricated.
• The ZVP/ZWP composite mainly consisted of ZWP and ZVP grains.
• The ZVP/ZWP composite had a TEC of −0.29 × 10^−7^ °C^−1^ in the range 25–500 °C.

## Graphical abstract

**Figure:**
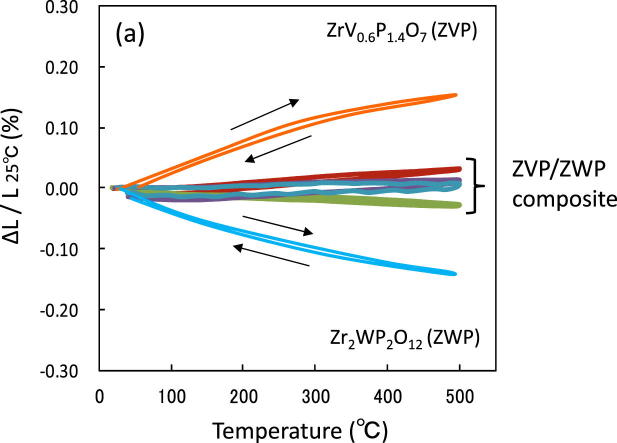

## 1 Introduction

Zero-thermal-expansion materials have recently received considerable attention for modern industries requiring high precision materials over wide temperature ranges. Compounds exhibiting negative thermal expansion over a wide temperature range are particularly useful for fabricating composites with zero or very low thermal expansion properties [1], [2], [3], [4], [5]. Cubic ZrW_2_O_8_ exhibits isotropic negative thermal expansion over a wide temperature range [6], [7], and various composites containing ZrW_2_O_8_ and metals [8], [9], [10], [11], ceramics [12], [13], and polymers [14], have been developed. However, the synthesis of a ZrW_2_O_8_ single phase requires quenching because of its pseudo-stable at room temperature [14], [15], which is disadvantageous for developing zero-thermal-expansion composites because the process causes microcracks.

Orthorhombic Zr_2_WP_2_O_12_ (ZWP) with a negative thermal expansion [16], [17], [18] can be synthesized by a solid-state method without quenching [18], [19], suggesting its potential use for fabricating zero-thermal-expansion materials. However, a high-temperature treatment is required to sinter ZWP because of its high melting point (ca. 1750 °C) [20]. Therefore, the addition of inorganic compounds that can be sintered at low temperatures and exhibit positive thermal expansion is needed to fabricate zero-thermal-expansion composites containing ZWP. However, there have been few reports on the fabrication of zero-thermal-expansion composites containing ZWP.

Negative thermal expansion above 150 °C for cubic P-substituted ZrV_2_O_7_ (ZVP), such as ZrV_1.2_P_0.8_O_7_ [21], [22], was suppressed compared with that of cubic ZrV_2_O_7_ [23], [24], [25]. Our previous study [22] reported that the positive thermal expansion property of ZVP increased with increasing P-substitution ratio. In particular, ZVP exhibited monotonous positive thermal expansion over a wide temperature range when a higher ratio of P was substituted for V [26]. Furthermore, ZrV_2_O_7_ easily melts at temperatures as low as 750 °C [27], suggesting the potential use of ZVP as a component in zero-thermal-expansion composites containing ZWP.

In this study, ZrV_0.6_P_1.4_O_7_ (ZVP) with a positive thermal expansion property and Zr_2_WP_2_O_12_ (ZWP) with a negative thermal expansion property were synthesized to fabricate a ZVP/ZWP composite with a near-zero thermal expansion property. The microstructures of the fabricated ZVP/ZWP composites were also investigated.

## 2 Experimental

For synthesis of Zr_2_WP_2_O_12_ powders, 0.05 M aqueous solutions of NH_4_H_2_PO_4_ (Wako Pure Chemical Ind., reagent grade) and (NH_4_)_10_W_12_O_41_ (Wako Pure Chemical Ind., reagent grade) were prepared to obtain a W/P molar ratio of 0.5, and a 0.05 M aqueous solution of ZrOCl_2_ (Wako Pure Chemical Ind., reagent grade) was also prepared. These solutions were then mixed to obtain a W/Zr molar ratio of 0.5 by stirring for 18 h at room temperature in air to produce a slurry. The obtained slurries were dried at 90 °C for 24 h to remove the solvent and were then heated at 900 °C for 4 h in air to synthesize the ZWP powders.

For synthesis of ZrV_0.6_P_1.4_O_7_ (ZVP) powders, 0.05 M aqueous solutions of NH_4_H_2_PO_4_ (Wako Pure Chemical Ind., reagent grade) and NH_4_VO_3_ (Wako Pure Chemical Ind., reagent grade) were prepared to obtain a P/V molar ratio of 1.4/0.6, and a 0.05 M aqueous solution of ZrO(NO_3_)_2_ (Wako Pure Chemical Ind., reagent grade) was also prepared. These solutions were then mixed to obtain a Zr/P molar ratio of 1/1.4 by stirring for 20 h at 60 °C in air to produce a slurry. The obtained slurries were dried at 90 °C for 24 h to remove the solvent and were then heated at 400 °C for 4 h in air to synthesize ZVP powders.

The synthesized ZWP and ZVP powders were then mixed in ethanol using ball milling for 18 h to obtain ZVP/ZWP volume ratios of 0.5/0.5, 0.53/0.47, 0.55/0.45, and 0.6/0.4. The prepared powder mixtures of ZWP and ZVP were shaped into a 5 mm × 5 mm × 12 mm compacts using uniaxial pressing at 98 MPa for 1 min, followed by cold isostatic pressing at 196 MPa for 1 min. The compacts were placed in a Pt boat in an electrical furnace and then sintered at 1000 °C for 20 h in air to fabricate ZVP/ZWP composites.

The crystalline phases of the synthesized powders and sintered bodies were examined by X-ray diffraction (XRD; RINT2000, Rigaku) with CuKα radiation. The microstructures of the fractured surfaces of the sintered bodies were examined by field-emission scanning electron microscopy (FESEM; S4100, Hitachi) and energy-dispersive X-ray spectrometry (EDX; Quantax400-125S). The thermal expansion properties of the sintered bodies in the range 25–500 °C were investigated by thermomechanical analysis (TMA; Thermoplus 8310, Rigaku) at heating and cooling rates of 5 °C/min. The bulk densities of the sintered bodies were measured by the Archimedes method with ion-exchanged water as the immersion medium.

## 3 Results and discussion

The thermal expansion coefficients (TECs) in the range 25–500 °C for the sintered bodies of ZWP and ZVP are shown in Table 1. The positive TEC of ZVP implies that the TEC of ZVP can cancel out the negative TEC of ZWP in the range 25–500 °C. Hence, the ZWP/ZVP composites were expected to exhibit near-zero thermal expansion over a wide temperature range of 25–500 °C. The TECs of the composites can be calculated by Eq. (1) [12], [16], [28]:

(1) $${\text{TEC}}_{\text{com}}={\text{TEC}}_{\text{ZWP}}\times {\text{V}}_{\text{ZWP}}/({\text{V}}_{\text{ZWP}}+{\text{V}}_{\text{ZVP}})+{\text{TEC}}_{\text{ZVP}}\times {\text{V}}_{\text{ZVP}}/({\text{V}}_{\text{ZWP}}+{\text{V}}_{\text{ZVP}})\text{.}$$

**Table 1:** Measured linear thermal expansion coefficients (×10^−6^ °C^−1^) of ZWP and ZVP sintered bodies and calculated thermal expansion coefficients (×10^−6^ °C^−1^) of ZVP/ZWP with a volume ratio of 0.53/0.47 in the range of 25 to 100, 200, 300, 400, and 500 °C .

	100 °C	200 °C	300 °C	400 °C	500 °C
ZWP	−4.55	−4.01	−3.53	−3.21	−2.92
ZVP	4.87	4.61	4.20	3.76	3.27
ZVP/ZWP = 0.53/0.47			(calculated)		
	−0.49	−0.31	−0.25	−0.24	−0.25

Here, TEC_com_, TEC_ZWP_, and TEC_ZVP_ are the TECs of the ZVP/ZWP composite, ZWP, and ZVP, respectively. V_ZWP_ and V_ZVP_ are the lattice volumes of ZWP and ZVP, respectively. The V_ZVP_/V_ZWP_ ratios for TEC_com_ = 0 were calculated by Eq. (1). The calculated V_ZVP_/V_ZWP_ ratios for TEC_com_ = 0 were 1.07 (25–100 °C), 1.15 (25–200 °C), 1.19 (25–300 °C), 1.17 (25–400 °C), 1.12 (25–500 °C). From the calculated results, the composite with a V_ZVP_/V_ZWP_ ratio of 1.127 (i.e., 0.53/0.47) was expected to show near-zero thermal expansion in the range 25–500 °C, as shown in Table 1.

**Fig. 1:**
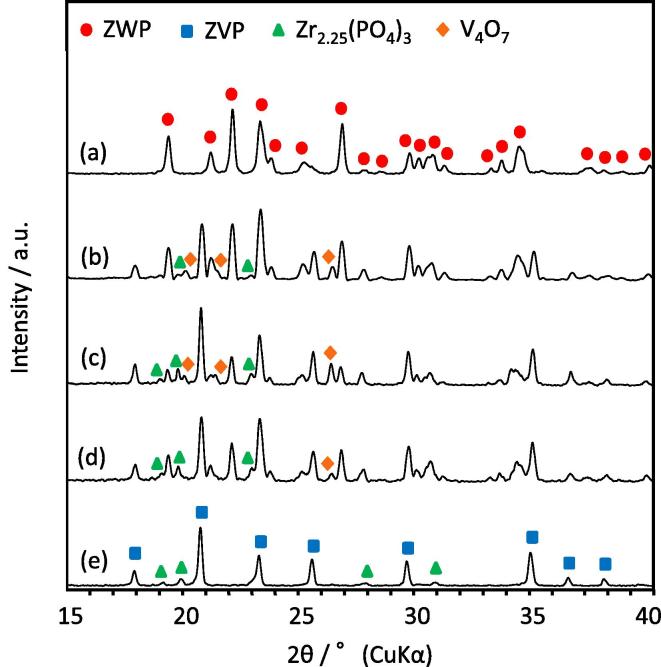
XRD patterns of ZrV_0.6_P_1.4_O_7_ (ZVP)/Zr_2_WP_2_O_12_ (ZWP) composites fabricated at V_ZVP_/V_ZWP_ ratios of (b) 0.6/0.4, (c) 0.55/0.45, and (d) 0.53/0.47, in addition to sintered bodies of (a) ZVP and (e) ZWP fabricated. All samples were sintered at 1000 °C for 20 h in air. (●) Zr_2_WP_2_O_12_ (ZWP), (■) ZrV_0.6_P_1.4_O_7_ (ZVP), (▲) Zr_2.25_(PO_4_)_3_, and (◆) V_4_O_7_.

Fig. 1 shows XRD patterns of the ZVP/ZWP composites with V_ZVP_/V_ZWP_ ratios of 0.53/0.47, 0.55/0.45, and 0.6/0.4 in addition to those of the sintered bodies of ZWP and ZVP. The XRD patterns confirm that the sintered composite mainly consisted of two phases of ZWP and ZVP in addition to a small amount of Zr_2.25_(PO_4_)_3_ (PDF #38–0017) and V_4_O_7_ (PDF #18–1452) impurities. The impurities were generated from production of a liquid phase [29], [30] derived from ZVP during the sintering process.

**Fig. 2:**
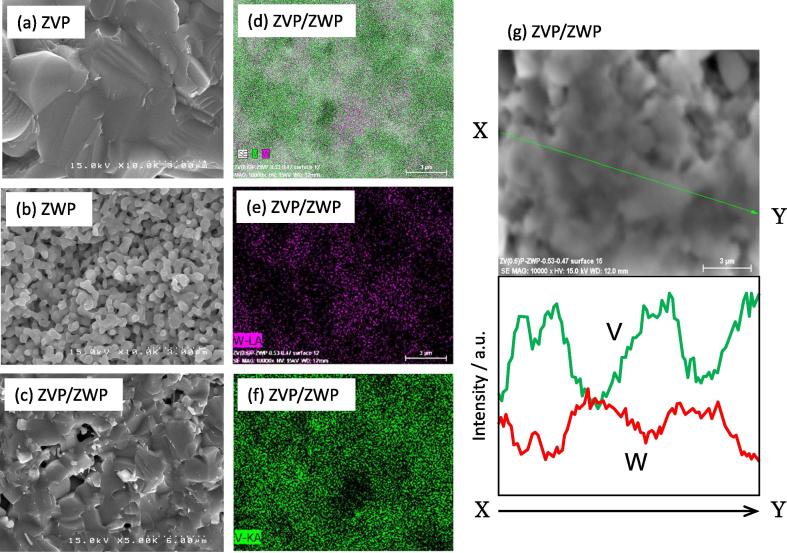
SEM images of fracture surfaces of sintered bodies of (a) ZrV_0.6_P_1.4_O_7_ (ZVP), (b) Zr_2_WP_2_O_12_ (ZWP), and (c) ZVP/ZWP composite fabricated at 1000 °C for 20 h in air and EDX composition maps (d) W and V, (e) W, (f) V, and (g) line analysis for ZVP/ZWP composite.

Fig. 2 shows SEM images of the fracture surfaces of the ZWP and ZVP sintered bodies and the composite fabricated at a V_ZVP_/V_ZWP_ ratio of 0.53/0.47 in addition to EDX composition maps and line analysis of the ZVP/ZWP composite with a V_ZVP_/V_ZWP_ ratio of 0.53/0.47. The ZVP sintered body fabricated at 400 °C was more densified compared with the ZWP sintered body fabricated at 900 °C because the melting point of ZVP was lower than that of ZWP. The porous structure of ZWP fabricated at 900 °C indicates that a higher temperature is necessary for densification of the ZWP sintered body, suggesting that the combination of ZVP and ZWP is effective for fabricating a sintered body. As shown in Fig. 2(c), the composite with a relative density of 82.3% had a porous microstructure intermediate between that of ZWP and ZVP. The microstructure implies that sintering of ZVP grains promoted sintering of the composite. The composition maps and line analysis confirm that the ZVP/ZWP composite had a microstructure consisting of ZVP and ZWP grains. The minor phases of Zr_2.25_(PO_4_)_3_ and V_4_O_7_ shown in Fig. 1 were thought to present around the ZVP grains because these phases were derived from the ZVP phase.

**Fig. 3:**
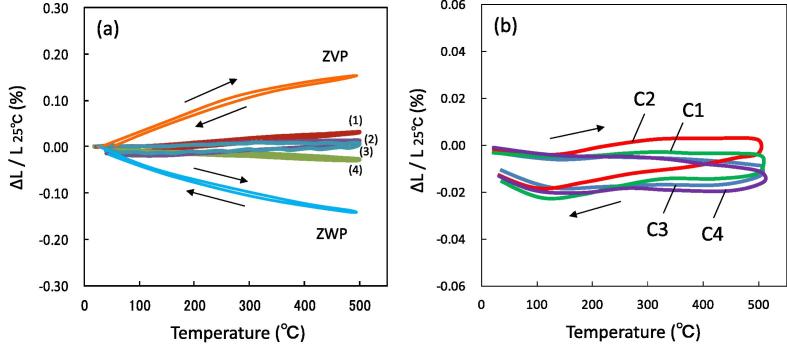
(a) Thermal expansion properties in the range 25–500 °C, in heating and cooling processes, for ZrV_0.6_P_1.4_O_7_ (ZVP)/Zr_2_WP_2_O_12_ (ZWP) composites fabricated at V_ZVP_/V_ZWP_ ratios of (1) 0.6/0.4, (2) 0.53/0.47, (3) 0.55/0.45, and (4) 0.5/0.5 in addition to ZVP and ZWP sintered bodies. (b) Cyclic thermal expansion properties of ZVP/ZWP composite fabricated at V_ZVP_/V_ZWP_ ratios of 0.53/0.47. C1: 1 cycle, C2: 2 cycles, C3: 3 cycles, C4: 4 cycles.

Fig. 3(a) shows the thermal expansion properties of the sintered bodies of ZVP, ZWP, and the ZVP/ZWP composites with V_ZVP_/V_ZWP_ ratios of 0.5/0.5, 0.53/0.47, 0.55/0.45, and 0.6/0.4. There were significant differences among the composite properties. The ZVP/ZWP composites with a V_ZVP_/V_ZWP_ ratio of 0.5/0.5 exhibited negative thermal expansion with a mean TEC of −5.59 × 10^−7^ °C^−1^ in the range 25–500 °C, and that with a V_ZVP_/V_ZWP_ ratio of 0.6/0.4 exhibited positive thermal expansion with a mean TEC of 6.64 × 10^−7^ °C^−1^ in the range 25–500 °C. However, the ZVP/ZWP composites with V_ZVP_/V_ZWP_ ratios of 0.53/0.47 and 0.55/0.45 exhibited very low thermal expansion in the range 25–500 °C. The ZVP/ZWP composite with a V_ZVP_/V_ZWP_ ratio of 0.53/0.47 exhibited a near-zero thermal expansion with a slight hysteresis and a mean TEC of −0.29 × 10^−7^ °C^−1^. The mean TEC of the ZVP/ZWP composite was similar to the calculated value in Table 1. The relative density of the ZVP/ZWP composite with a V_ZVP_/V_ZWP_ ratio of 0.53/0.47 was 82.3% and that of the ZVP/ZWP composite with a V_ZVP_/V_ZWP_ ratio of 0.5/0.5 was 83.2%. These results suggest that the thermal expansion properties of the composites were mainly influenced by the ZVP/ZWP ratio and not influenced by the porosity of the composite. Fig. 3(b) shows the cyclic thermal expansion property of the ZVP/ZWP composite with a V_ZVP_/V_ZWP_ ratio of 0.53/0.47. No significant differences were observed among the thermal expansion properties regardless of the number of cycles. Thus, a near-zero-thermal-expansion material was successively fabricated, containing ZWP with negative thermal expansion and ZVP with positive thermal expansion.

## 4 Conclusions

ZrV_0.6_P_1.4_O_7_ (ZVP) exhibiting positive thermal expansion was synthesized and combined with ZWP exhibiting negative thermal expansion to fabricate a near-zero-thermal-expansion material. Compacts of the calcined ZWP and ZVP powders with V_ZVP_/V_ZWP_ ratios of 0.5/0.5, 0.53/0.47, 0.55/0.45, and 0.6/0.4 were fabricated and sintered at 1000 °C for 20 h in air. Sintering of the ZWP/ZVP composites progressed well compared with that of ZWP. In addition, the composite fabricated at a V_ZVP_/V_ZWP_ ratio of 0.53/0.47 had a microstructure with a relative density of approximately 83% and exhibited reversible near-zero-thermal-expansion with a TEC of −0.29 × 10^−7^ °C^−1^ in the range 25–500 °C.
